# Exploring the potential of *Rhizopus oryzae* AUMC14899 as a novel endophytic fungus for the production of l-tyrosine and its biomedical applications

**DOI:** 10.1186/s12934-023-02041-1

**Published:** 2023-02-20

**Authors:** Nessma A. El-Zawawy, Sameh Samir Ali, Hoda S. Nouh

**Affiliations:** Botany Department, Faculty of Science, Tanta University, Tanta, 31527 Egypt

**Keywords:** Endophytic fungi, *Opuntia ficus-indica*, Antibacterial activity, Antibiofilm activity, l-tyrosine

## Abstract

**Background:**

A significant threat to the public's health is the rise in antimicrobial resistance among numerous nosocomial bacterial infections. This may be a detriment to present initiatives to enhance the health of immune-compromised patients. Consequently, attention has been devoted to exploring new bioactive compounds in the field of drug discovery from endophytes. Therefore, this study is the first on the production of l-tyrosine (LT) as a promising bio-therapeutic agent from endophytic fungi.

**Results:**

A new endophytic fungal isolate has been identified for the first time as *Rhizopus oryzae* AUMC14899 from *Opuntia ficus-indica* (L.) and submitted to GenBank under the accession number MZ025968. Separation of amino acids in the crude extract of this fungal isolate was carried out, giving a higher content of LT, which is then characterized and purified. LT exhibited strong antibacterial and anti-biofilm activities against multidrug-resistant Gram-negative and Gram-positive bacteria. The recorded minimum inhibitory concentration (MIC) values ranged from 6 to 20 µg/ml. In addition, LT caused a strong reduction in biofilm formation and disrupted the preformed biofilm. Moreover, results indicated that LT supported cell viability, evidencing hemocompatibility and no cytotoxicity.

**Conclusion:**

Our findings suggest that LT has potential as a therapeutic agent due to its potential antibacterial, anti-biofilm, hemocompatibility, and lack of cytotoxic activities, which may also increase the range of therapy options for skin burn infections, leading to the development of a novel fungal-based drug.

**Supplementary Information:**

The online version contains supplementary material available at 10.1186/s12934-023-02041-1.

## Background

Burn wounds are considered a huge public health issue all over the world, especially in developing countries [1–3]. Due to the immunosuppressive consequences of burn injury, hospitalized patients in burn care units are at a higher risk for nosocomial infections [4, 5]. Nosocomial infections are among the most common problems that impact hospitalized patients, contributing to increased morbidity and mortality [6, 7]. Approximately 50–75% of morbidity in hospitalized burn patients is due to microbial infections [5]. *Staphylococcus aureus* and *Pseudomonas aeruginosa* are the most common bacteria in burn wounds [8]. Burns caused by both *P. aeruginosa* and *S. aureus* have resulted in a chronic, non-healing wound [9, 10]. The presence of host microbiota-associated pathogens in burns could lower the efficiency of burn healing [11]. In post-burn infections, where antibiotic resistance frequently leads to treatment failure and chronic infections, biofilm is also regarded as a key virulence component [4, 12]. In order to adhere to surfaces and protect pathogens from host immune reactions and antibiotics, bacteria form organized communities called biofilms that are enclosed in a polymeric matrix [13]. Most isolated bacteria from burn patients frequently develop biofilms on the ulcerated sections of burn wounds [4]. Microorganisms have created a variety of strategies to counter antimicrobial drugs, such as reducing cellular permeability to decrease drug penetration, enzymatically breaking down antimicrobial compounds, and forming biofilms [14, 15].

Multidrug resistance (MDR) is not a recent phenomenon, but it is a critical health issue today [16–18]. Hundreds of thousands of deaths worldwide are due to the rising issue of MDR each year [19]. It is predicted that, if the problem is not adequately handled, more than 10 million annual deaths will be caused by MDR pathogens by the year 2050, surpassing cancer-related deaths [20]. Recent reports have documented an increasing proportion of MDR *Staphylococcus aureus* and *Pseudomonas aeruginosa* associated with wound infections [4, 5]. Worldwide, MDR bacteria cause serious infections as a result of bacterial adaptation that includes the development of mutations, transformation, conjugation, transposition, and transduction [21]. Increasing MDR has generated scientific interest to discover novel antimicrobial agents with broad spectrum activities to complement and/or replace conventional antibiotics [5, 15, 22].

Medicinal plants play a significant role in the field of drug discovery since they are regarded as a promising source of natural compounds for medical purposes [23, 24]. Additionally, they operate as hosts for several endophytes, which are known to produce a variety of bioactive substances [25–27]. Endophytic microorganisms showed a wide range of biological activity, including anticancer, antibacterial, antifungal, immunomodulatory, antioxidant, and textile dyeing activities [28–30]. Endophytic fungi are a significant part of the plant microbiota that is present throughout the plant tissues without harming the host. They serve a crucial function in protecting the host from a variety of biotic stresses that have been shown to be associated with their antimicrobial activity [31]. Alkaloids, steroids, and amino acids have been extracted and identified as the secondary metabolites responsible for different biological activities shown by fungal endophytes [10, 32]. Numerous studies on endophytic fungi have shown that, while some of them produce secondary metabolites that are comparable to those produced by their host plants, others produce a wide range of substances, making them a promising source of novel substances that are more bioactive than those produced by their hosts [33–35]. *Opuntia ficus-indica* (L.), often known as prickly pear, is well known for its wide range of medical benefits as an antioxidant, an anti-inflammatory agent, and in the treatment of ulcers [36, 37]. Interestingly, amino acids that have been isolated from its cactus have several biological activities [38]. Amino acids may be used as anti-biofilm agents and pharmacological excipients in future therapeutic procedures. There have been numerous reports of free amino acids and peptides having antibacterial properties [39, 40]. Additionally, d-amino acids have been utilized to improve the antimicrobial efficacy (in vitro) of approved medications [41]. The link between l-amino acids and anti-biofilm activity has also been investigated [42].

l-tyrosine (LT) has attracted increasing attention due to its special functions in humans and animals. It is used as a nutritional supplement to treat depression in patients [43]. It is also a key precursor of neurotransmitters like dopamine and thyroxine, which control nerve signaling and nervous system development [44]. LT, as an aromatic platform compound, can be used to synthesize high-value drugs such as resveratrol and tyrosol [45]. Chemical enzymatic production is the traditional method of producing LT. Due to its high cost, low extraction yield, and heavy environmental pollution, this method is not suitable for large-scale industrial production [46]. Nowadays, due to increasing attention to environmental protection and sustainable development, LT production via microbial fermentation has become more interesting for researchers. Production of LT through microbial fermentation is a workable alternative with the development of synthetic biology [47].

Treatment of wound burn infections with conventional antibiotics is inadequately effective against MDR pathogens, making alternative or complementary approaches for combating drug resistant pathogens inhabiting burn wounds highly desirable. To this end, an increasing number of novel compounds are being screened from endophytic fungi due to their abundant reservoir of bioactive metabolites. Therefore, the purpose of this study is to investigate the potential of *Rhizopus oryzae* AUMC14899 as a novel endophytic fungus for the production of LT and explore its biomedical applications. The findings of this study open up a new avenue for screening and characterizing new bioactive metabolites from endophytic fungi isolated from *Opuntia ficus-indica* as alternative antimicrobial agents against skin burn infections.

## Materials and methods

### Plant collection and isolation of endophytic fungi

**Fig. 1:**
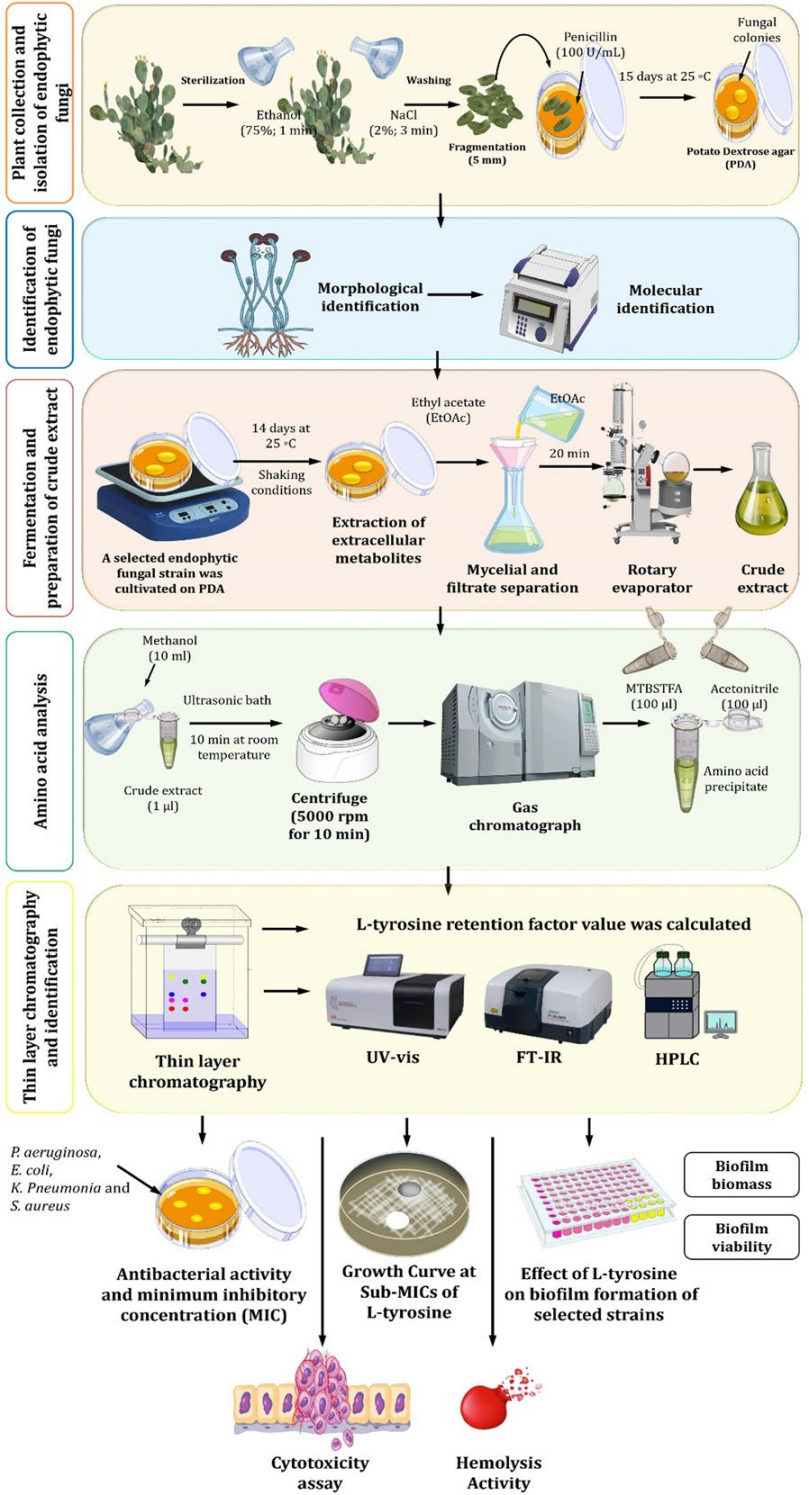
Experimental design presented in this study to explore the potential of l-tyrosine (LT) extracted from endophytic fungi *Rhizopus oryzae* AUMC14899 and its therapeutic applications

Cladodes of *O. ficus-indica* (L.) Mill. (Cactaceae) were randomly collected from Berket Al Sabaa along the train road of Cairo‐Alexandria, Monufiya Governorate, Egypt, in March 2021 (Additional file 1: Fig. S1). The samples were delivered to the laboratory within 48 h by placing them in a plastic bag inside an icebox. Soil was removed from samples by rinsing them under running tap water. The samples were sterilized sequentially by washing with 75% ethanol for 3 min, 10% sodium hypochlorite for 10 min, and 0.1% mercuric chloride for 2 min. Finally, the samples were rinsed in sterile distilled water four to six times [48]. Sterilized cladodes were cut into 5-mm long fragments using a flame-sterilized scalpel under aseptic conditions [49]. After that, three fragments were placed separately on potato dextrose agar plates (PDA, Sigma–Aldrich, St. Louis, Missouri) supplemented with penicillin (100 U/ml) (Sigma/USA) [10]. The plates were incubated for up to 15 days at 25 °C. After incubation, culture purity was assessed, and fungal colonies were stored in glycerol at − 80 °C for further use (Fig. 1).

### Identification of endophytic fungi

The chosen endophytic fungal isolate used in this study was identified using conventional morphological features. Aerial mycelium and density were noted as colony characteristics. Micro-morphological properties were examined under a light microscope (Olympus CX51, Japan) as previously described [50].

Molecular techniques were used to confirm the identification of a chosen endophytic fungal isolate by sending cultures to the Molecular Biology Research Unit, Assiut University, for DNA extraction using the patho-gene-spin DNA/RNA extraction kit, which was made available by the Korean company Intron Biotechnology. Samples of fungal DNA were submitted to SolGent Company in Daejeon, South Korea, for PCR and rRNA gene sequencing. PCR was performed using ITS1 (forward) and ITS4 (reverse) primers, which were incorporated into the reaction mixture. Primers have the following composition: ITS1 (5′-TCC GTA GGT GAA CCT GCG G-3′), and ITS4 (5′- TCC TCC GCT TAT TGA TAT GC -3′). The dNTPs were added to the reaction mixture together with the same primers to sequence the purified PCR result [51]. The collected sequences were examined using the Basic Local Alignment Search Tool (BLAST) accessible on the National Center for Biotechnology Information (NCBI) website. The obtained sequences were subjected to BLAST analysis with the deposited sequences in the NCBI database to find the homology with the closest related organisms. The neighbor-joining phylogenetic tree for the ITS rRNA gene was constructed using MEGA X software [52].

### Fermentation and preparation of crude extract

A selected endophytic fungal strain was cultivated in potato dextrose broth (PDB) for 14 days at 25 °C under static conditions [53]. After fermentation, an ethyl acetate solvent was used to extract the extracellular secondary metabolites [54]. In a funnel, broth culture was filtered through Whatman No. 1 filter paper in order to separate the mycelia. The filtrate was three times extracted with an equivalent amount of ethyl acetate, and then the mixture was allowed to stand for 20 min. The organic solvent phase was next separated and concentrated under reduced pressure using a rotary evaporator. The resulting crude extract was then kept at a temperature of − 20 °C for further use (Fig. 1).

### Extract derivatization for amino acid separation

A fungus crude extract solution was prepared as follows: Ten milliliters of methanol were used to extract one microgram of the extract in an ultrasonic bath at room temperature for 10 min. The resulting solution was centrifuged at 5000 rpm for 10 min at 25 °C. Under nitrogen gas, 500 µl of supernatant was evaporated to dry the residue. The resultant precipitate was dissolved in 100 µl of acetonitrile and 100 µl of *N*-(t-butyldimethylsilyl)-N109 methyltrifluoroacetamide (MTBSTFA) reagent. In a glycerol bath, this solution was heated at 100 °C for 2.5 h. The tested solution was then injected into a gas chromatograph at a volume of 1 µl. Similar to this, standard solutions were made by taking 100 µl of the l-amino acid standards mixture (reference samples of alanine, serine, valine, threonine, leucine, isoleucine, proline, aspartic acid, glutamic acid, lysine, methionine, phenylalanine, and tyrosine) and drying them under nitrogen gas to dry residue. Next, 100 µl of acetonitrile and 100 µl of MTBSTFA were added. The resulting mixture was heated for 2 h at 100 °C in a glycerol bath.

### Amino acid analysis

The analysis was performed at the Scientific Research Centre and Measurement, Tanta University, Tanta, Egypt, using a gas chromatograph (SHIMADZU GC–MS-QP2010) according to Mykhailenko et al. [55]. Rxi-5 ms capillary column (30 m long, 0.25 mm outer diameter, and 0.25 m liquid-stationary phase thickness) was used to separate amino acids using helium as the carrier gas. The liquid stationary phase included 5% diphenyl and 95% polysiloxane.

### Identification and characterization of LT

Thin layer chromatography (TLC) was used for the separation of amino acids, especially LT due to its higher content. A silica gel G60 F_254_ plate (Merck, Germany) was used as a stationary phase. The mobile phase was 2-methyl-2-propanol, ethanoic acid, and water (25:6:10). A reference standard, LT (Merck, Darmstadt, Germany), was used. The separation method was applied according to Li et al. [56]. After the separation process, the R_f_ (retention factor) value of LT from fungal crude extract was calculated and compared to the standard one.

After scratching the LT band from TLC, ultraviolet (UV) analysis took place to confirm the purification of the selected LT using the Perkin Elmer Lambda 4B UV–vis spectrophotometer [57]. Fourier transform-infrared spectroscopy (FT-IR) analysis using an IR spectrophotometer (Perkin-Elmer 1430) was done to detect all the chemical functional groups present in LT. Numerous modes of vibration have been reported by FT-Raman spectroscopy using a Bruker RFS 27 spectrometer and variously assigned [58]. To confirm the functional groups identified, the ^13^C and ^1^H NMR spectra of LT were recorded using a JEOL GSX 400 instrument at 30 °C [59]. Thermal analysis was achieved through a differential scanning calorimetric curve (DSC) to find the degree of decomposition of LT. Moreover, high performance liquid chromatography (HPLC) was used to confirm the identification of LT by using Thermo Scientific Dionex Ultimate 3000 UHPLC at Pharmaceutical Service Center, Faculty of Pharmacy, Tanta University, Tanta, Egypt [46].

### Determination of antibacterial activity and minimum inhibitory concentration

Six MDR bacterial strains, isolated from burn wound infections, were used in this study. These strains were identified as *P. aeruginosa* PA-02, *P. aeruginosa* PA-09, *Escherichia coli* EC-03, *Klebsiella pneumonia* KP-01, *S. aureus* SA-03, and *S. aureus* SA-04, as we previously reported [5, 6, 57]. Screening of antibacterial activity of LT against the tested bacterial strains was estimated by the agar well diffusion method, as described previously in detail with a minor modification [60]. Culture suspensions of bacterial strains were prepared to contain 10^6^ CFU/ml, and the agar plates were inoculated with 100 µl of the broth cultures of the tested strains. LT was dissolved in 1% pure dimethyl sulfoxide (DMSO; Sigma-Aldrich, St. Louis, Missouri, USA) to a final concentration of 100 µg/ml. Twenty microliters of different concentrations of LT (10, 20, 30, 40, and 50 µg/ml) were pipetted into a hole (5 mm width) made in the center of the agar plate. DMSO (1%) was used as a negative control, and streptomycin (10 µl) was used as a positive control [23]. The minimum inhibitory concentration (MIC) was determined by the microdilution method using resazurin dye [61]. The growth was observed through the change in color of the resazurin dye from pink to red, which indicated the presence of living cells. The presence of a dark blue color showed the complete inhibition of bacterial growth (Fig. 1). The MIC was calculated as the lowest sample concentration that prevented bacterial growth. Sub-minimal inhibitory concentrations (Sub-MICs) were selected for the assessment of the biofilm prevention efficacy of LT against the tested MDR bacterial strains.

### Growth curve at sub-MICs of LT

An aliquot (100 µl) of overnight culture suspensions (1 × 10^6^ CFU/ml) of bacterial strains were added to an equal volume of LT dissolved in 1% DMSO to obtain 1/2, 1/4, 1/8, and 1/16 × MICs in MHB and were incubated at 37 °C for 24 h in a shaker incubator at 120 rpm (Fig. 1). The turbidity of the bacterial cultures (at OD_660_ nm) was assessed after 24 h. Maximum bacterial growth was measured using bacteria cultured in wells without LT as positive controls, while 1% DMSO was used as a negative control [62].

### Biofilm production

Using the microtitre plate (MTP) technique, the biofilm production of the six chosen strains was quantified [63]. Trypticase soy broth (TSB) containing 1% glucose was used to inoculate all of the investigated organisms that were isolated from fresh agar plates. For 24 h, broths were incubated at 37 °C. Then, fresh medium was added to the cultures at a 1:10 dilution. Two hundred microliters of the diluted cultures were added to each well of sterile 96-well microtitre plates. The negative control wells were filled with sterile broth. The plates were incubated for 24 h at 37 °C. Following incubation, each well's contents were taken out by light tapping. The wells were washed four times with 0.2 ml of phosphate buffered saline (pH 7.2). This eliminated bacteria that were floating around. Bacterial biofilm adhered to the wells was preserved with (2%) sodium acetate and stained with 0.1% crystal violet. Glacial acetic acid (33%) was used to remove extra discoloration, and the plates were kept to dry. At a wavelength of 570 nm, a micro ELISA autoreader (model EMR500, Labomed, USA) was used to measure the optical density of a stained adherent biofilm. The experiment was carried out in triplicate, and biofilm production was interpreted [64].

### Effect of LT on biofilm production and bacterial viability

The biofilm staining technique was used to evaluate the inhibitory efficacy of LT on biofilm development in vitro [65]. Bacterial suspensions (5 × 10^6^ cells/ml) of the tested strains were prepared in TSB supplemented with 0.5% glucose. Then, 100 μl per well was transferred to microtiter plates with 100 μl of different concentrations (sub-MICs) of LT ranging from 0.18 to 3 µg/ml for PA-02 and from 0.93 to 7.5 µg/ml for SA-04. The bacterial strains PA-02 and SA-04 were selected as the most biofilm producers. Wells filled with 100 μl of TSB mixed with 100 μl of DMSO without LT, and wells filled with TSB (100 μl) and DMSO (100 μl) without bacteria were used as negative controls. The supernatant was gently removed after 24 h of incubation at 37 °C. Then, 200 µl of 1X phosphate-buffered saline (PBS) was used to rinse the wells twice. The plates were let to dry at 60 °C for 30 min, and 200 µl of 0.1% crystal violet was added for 15 min to stain the biofilms. An aliquot (200 µl) of 96% ethanol was added and then washed with 200 µl of 1X PBS. The absorbance was then measured at 595 nm after 20 min at room temperature (Fig. 1). The formula used to determine the percentage of biofilm mass reduction was [(A*c*-A*t*)/A*c*] × 100, where A*c* represents the OD_595_ for the control well and A*t* represents the OD_595_ for the biofilm in the presence of LT.

Biofilms were allowed to develop in each well of a 96-well microtiter plate. Planktonic cells were gently aspirated off the plate after 24 h, and 200 μl of 1X PBS was then used to wash the plate. For PA-02 and SA-04, 200 µl of LT were added, with concentrations ranging from 6 to 24 µg/ml and from 15 to 60 µg/ml, respectively. The plate was then incubated for 24 h at 37 °C. A broth containing 200 μl of 1% DMSO was used to incubate untreated cells. After the incubation, the biofilm biomass was measured using crystal violet staining. Following a 24 h incubation period, biofilms in each well were allowed to develop before being treated as previously mentioned. XTT [2,3-bis (2-methyloxy-4-nitro-5-sulfophenyl)-2H-tetrazolium-5-carboxanilide] and *N*-methyl dibenzopyrazine methyl sulphate (Roche Diagnostics) were mixed together and added to each well after LT treatment in a volume of 150 µl. The biofilm metabolic activity was determined by measuring the absorbance value at 490 nm after incubation in the dark for 40 min at 37 °C. The viability results were examined in relation to control samples treated with 1% DMSO [66]. Each data point was expressed as the mean standard deviation of three distinct experiments.

Untreated and treated biofilms of tested bacterial strains with LT at 1/2 MIC concentrations (3 and 7.5 µg/ml for PA-02 and SA-04, respectively) were fixed in 2.5% glutaraldehyde in 0.1 M PBS (pH 7.4) at 4 °C for 2 h [67]. The fixed samples were washed three times with PBS. The samples were post-fixed in 1% osmic acid for 30 min, then dehydrated for 30 min using a range of ethyl alcohol concentrations (30, 50, 70, 90%, and absolute alcohol) infiltrated with acetone. The samples were mounted on aluminum stubs and dried in a Critical Point Dryer (Tousimis Autosamdri - 815 Coate) before being coated with gold in an SPI- Module (Sputter Carbon/Gold Coater). The samples were examined at the electron microscopy unit in the Faculty of Agriculture at EL-Mansoura University, El-Mansoura, Egypt, using a scanning electron microscope (JSM-6510 LV, JEOL, Japan).

### Cytotoxicity assay

The evaluation of purified LT cytotoxicity was carried out using non-tumor and tumor cell lines, namely HSF (human skin fibroblasts) and A-431 (human epidermoid skin carcinoma), as described previously [68]. Different concentrations of LT (0.5–500 µg/ml) were applied to cells compared to untreated cells (10 µl/well). The results were presented as half inhibitory concentrations (IC_50_), which is the sample concentration at which cell growth was 50% inhibited, and were represented in µg/ml.

### Hemolysis activity

The hemocompatibility test was carried out to assess the degree of RBC destruction in the presence of LT when it comes into contact with human erythrocytes [69]. The PBS was used to diffuse LT at various doses (0, 10, 20, 30, 40, and 50 µg/ml). The positive and negative controls were Triton X-100 and PBS, respectively. At 545 nm, the free hemoglobin content was determined spectrophotometrically as an indicator of hemolysis. Hemolysis (%) = [(AT − AN)/(AP − AN)] * 100, where AT, AN, and AP are the absorbance values of the tested sample, the negative control, and the positive control, respectively.

### Statistical analysis

Each experiment was performed three times, and GraphPad Prism 8.0 Software (San Diego, Canada) was used to analyze the findings. A one-way analysis of variance (ANOVA) test was used to determine the statistical significance of the results at a *P* value ≤ 0.05. The values represent the mean of three independent replicates, with error bars representing the standard deviation of differences that are statistically significant at ^*^P ≤ 0.05, ^**^P ≤ 0.01, ^***^P ≤ 0.001, and ^****^P ≤ 0.0001, while *P* > 0.05 is non-significant (ns).

## Results

### Isolation and identification of the endophytic fungal isolate

**Fig. 2:**
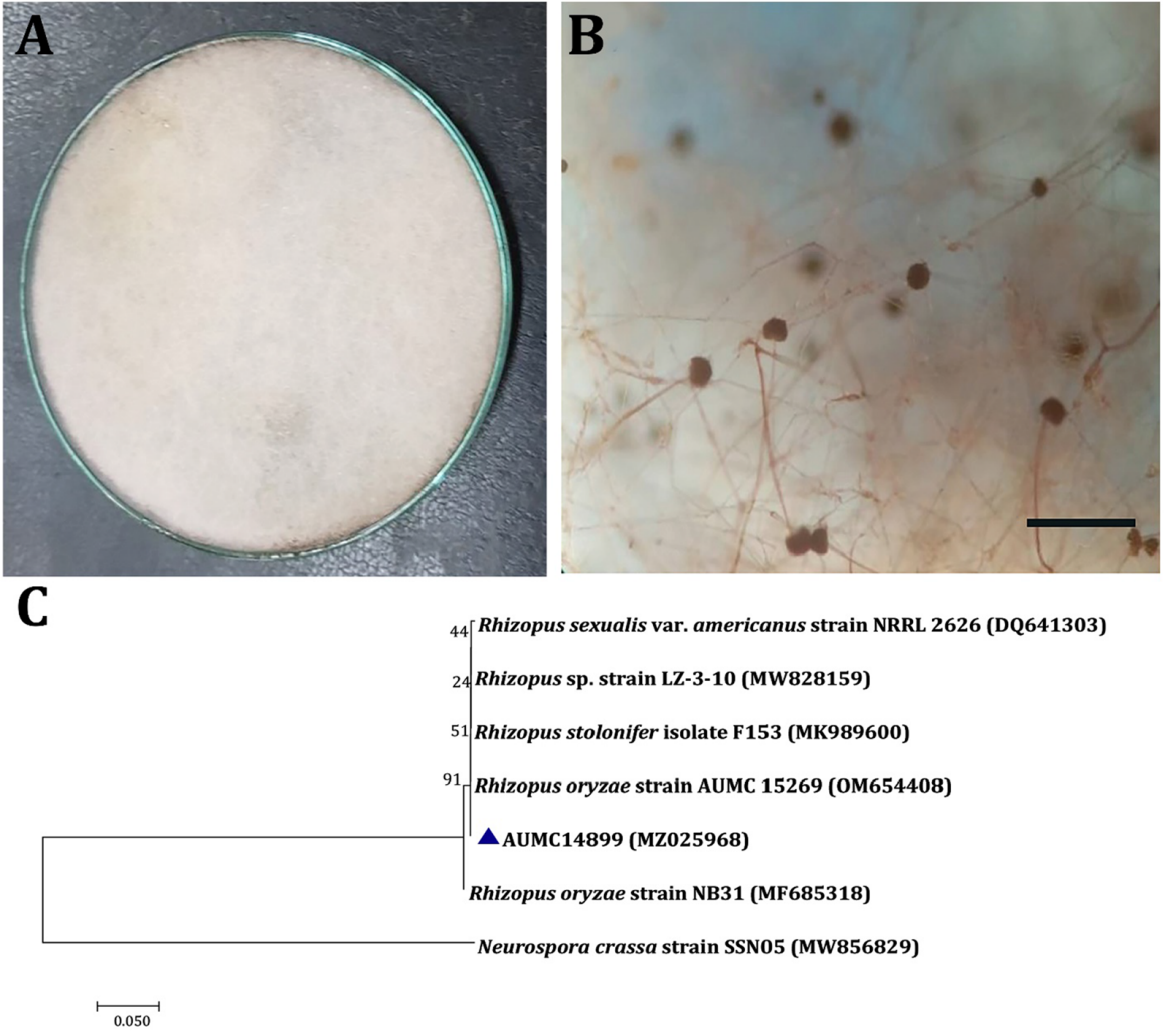
Morphological and molecular identification of *Rhizopus oryzae* AUMC14899. Colony on potato dextrose agar at 25 °C for 1 week (**A**). Sporangiophore and sporangia at ×40 magnification and scale bar represent 5 µm (**B**). Phylogenetic relationship between *Rhizopus oryzae* strain AUMC14899 and other fungal strains retrieved from database, based on partial 18S rRNA gene sequences. The phylogenetic tree was constructed using the neighbor-joining method with the Tamura 3-parameter model with 1000 bootstrap replicates. Value mentioned at nodes is bootstrap values and our strain is mentioned against blue triangle (**C**)

Colonies of selected isolates grew on PDA to 80–85 mm after 8 days at 25 °C in the dark. The fungal colonies began as white and cottony, changed to being extensively sporangia-speckled, and eventually became brownish-grey to blackish-grey (Fig. 2A). Sporangiospores are irregular, numerous, sub-globose or oval, while sporangiophores were straight, non-septate, simple or branched. Also, sporangia were globose, white at first, and then turned black (Fig. 2B). This isolate of fungus was recognized as *R. oryzae* based on morphological findings. The evolutionary history of *R. oryzae* AUMC14899 was inferred using the neighbor-joining method (Fig. 2C). *R. oryzae* AUMC14899 (MZ025968) showed 100% identity to *R. oryzae* strain AUMC 15269 (OM654408).

### Fermentation and amino acid analysis

After cultivation of *R. oryzae* isolate AUMC14899, the produced amino acids were assessed in its crude extract. Amino acids were identified by comparing the retention times of selected amino acids in GC–MS chromatograms. Quantitative analysis was performed using calibration curves built with standard solutions. Amino acid analysis resulted in the identification of seven essential amino acids and nine non-essential amino acids, which represented 38.39% and 61.61% of the total amino acids, respectively (Table 1). l-methionine and LT were detected as major essential (11.88%) and non-essential (17.92%) amino acids, respectively.

**Table 1:** Total l-amino acids of crude extract of *Rhizopus oryzae* strain AUMC14899

Amino acids	Molecular weight (g/moL)	RT (min)	Relative percentage (%) of total amino acids	RT^*^	Chemical formula
l-Aspartic	133.1	10.13	5.26	10.21	C_4_H_7_NO_4_
l-Threonine	119.119	13.08	3.34	13.12	C_4_H_9_NO_3_
l-Serine	105.09	13.93	7.83	13.98	C_3_H_7_NO_3_
l-Glutamic acid	147.13	15.52	2.33	15.45	C_5_H_9_NO_4_
l-Proline	115.13	22.43	6.96	22.41	C_5_H_9_NO_2_
l-Glycine	75.067	22.52	7.57	22.54	C_2_H_5_NO_2_
l-Alanine	89.09	24.13	4.29	24.18	C_3_H_7_NO_2_
l-Valine	117.151	30.73	3.88	30.77	C_5_H_11_NO_2_
l-Methionine	149.21	35.52	7.88	35.49	C_5_H_11_NO_2_S
l-Isoleucine	131.17	38.15	2.88	38.2	C_6_H_13_NO_2_
l-Leucine	131.17	39.90	3.26	39.85	C_6_H_13_NO_2_
l-Tyrosine	181.19	42.90	21.92	42.88	C_9_H_11_NO_3_
l-Phenylalanine	165.19	44.78	5.92	44.81	C_9_H_11_NO_2_
l-Histidine	155.15	50.20	3.97	50.21	C_6_H_9_N_3_O_2_
l-Lysine	146.19	53.20	7.23	53.05	C_6_H_14_N_2_O_2_
l-Arginine	174.20	61.48	5.48	61.45	C_6_H_14_N_4_O_2_

RT: Retention time of amino acids in crude extract; RT^*^: Retention time of standard amino acids

### Characterization of LT

**Fig. 3:**
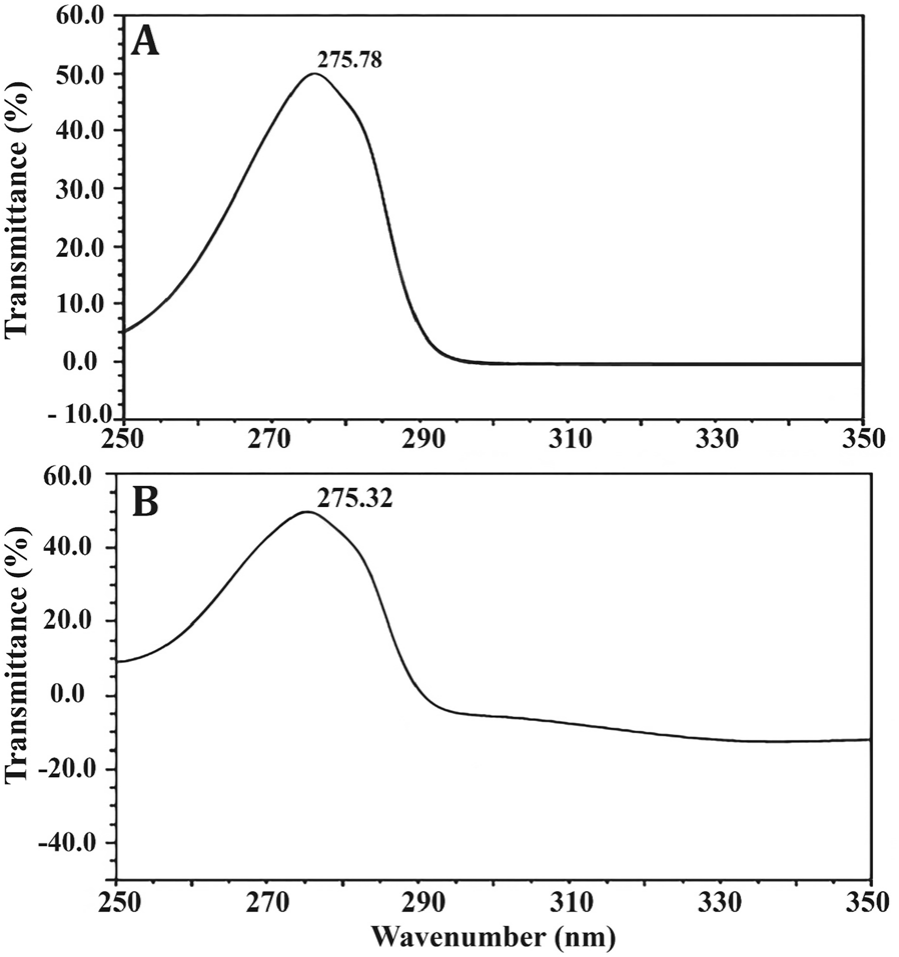
UV–Vis absorption spectrum of l-tyrosine extracted from *Rhizopus oryzae* strain AUMC14899. l-tyrosine standard (**A**). Isolated l-tyrosine from the endophytic fungus (**B**)

**Fig. 4:**
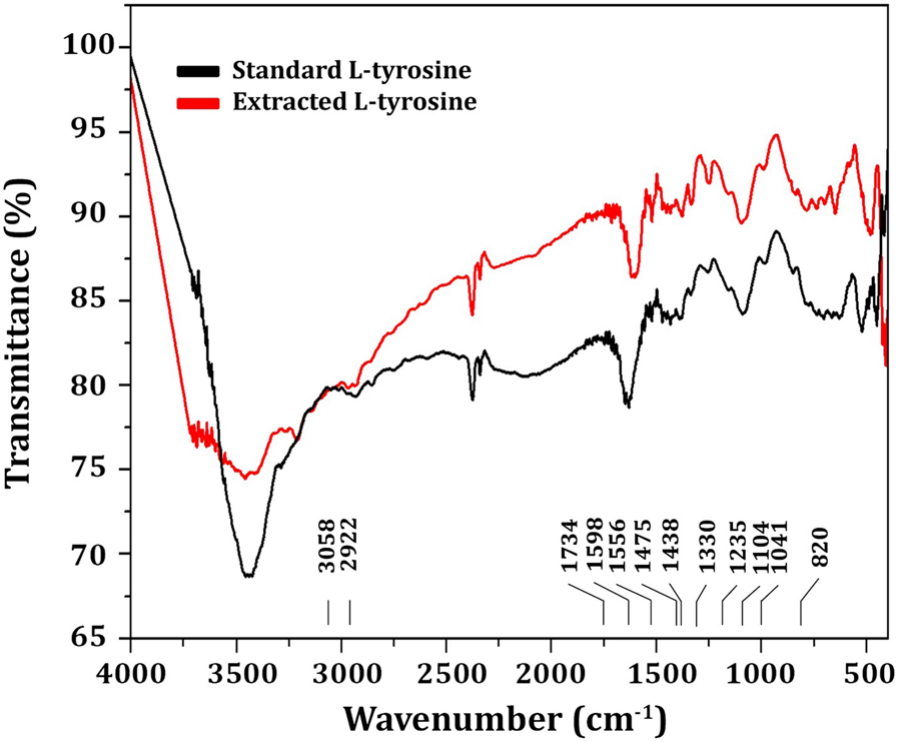
FTIR spectrum of l-tyrosine extracted from *Rhizopus oryzae* strain AUMC14899

**Fig. 5:**
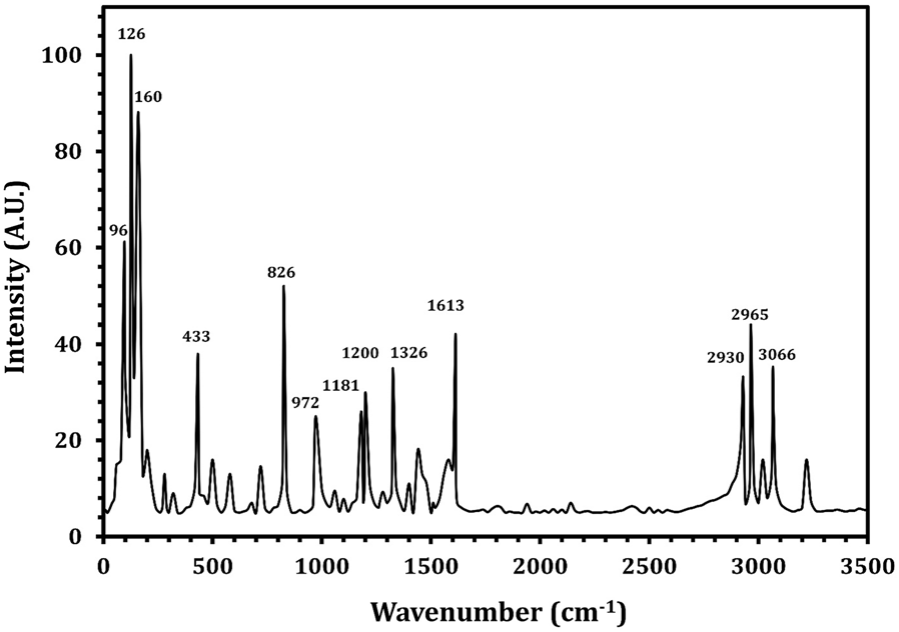
FT-Raman spectrum of l-tyrosine extracted from *Rhizopus oryzae* strain AUMC14899

The most active amino acid of the crude extract of *R. oryza* was LT, which was separated by TLC, and the results indicated that isolated LT has an R_f_ of 1.2, exactly similar to the R_f_ value of standard LT (Additional file 1: Fig. S2). The purified LT recovered from TLC was confirmed by UV spectrum analysis (Fig. 3), and the findings showed a strong peak at 275.32 nm (Fig. 3B) that is predominantly made up of the aromatic side-chains of tyrosine as in standard (Fig. 3A). The purified LT was characterized using an FTIR spectrum, and the functional group changes between the extracted LT and the standard LT are depicted in Fig. 4. The FTIR absorption peaks in the spectra of LT and their assignments are given in Table 2. The band at lower frequency 820 cm^−1^ is refer to NH_2_ wagging_._ The C–H in plane bending mode is assigned at 1041 cm^−1^. The CH_2_ bending mode is observed at 1438 cm^−1^. The C–C stretching mode occurred at 1734 cm^−1^. Most of these vibrations are determined in the FT-Raman spectrum (Fig. 5), the sharp peaks are due to the O–H stretch of –COOH and phenolic O–H and the N–H stretch of NH_3_^+^, which are detected between 2900 and 3070 cm^−1^. Below 3000 cm^−1^_,_ the CH_2_ vibrations are observed in the Raman spectra.

**Table 2:** FTIR absorption peaks in the spectra of LT and their assignments

Range of wave number (cm^−1^)	Assignment
3058	C–H asym. stretching (aliphatic)
2922	CH_2_ sym stretching
1734	C=C stretching
1598	NH_2_ scissoring
1556	C–C–C stretching
1475	C–H in plane bending in ring
1438	CH_2_ scissoring
1475	C–H in plane bending in ring
1330	Combination of C–C–C stretching and Phenolic OH
1235	Phenolic OH stretch with ring carbon
1104	C–N stretching
1041	C–H in plane bending
820	NH_2_ wagging

**Fig. 6.**
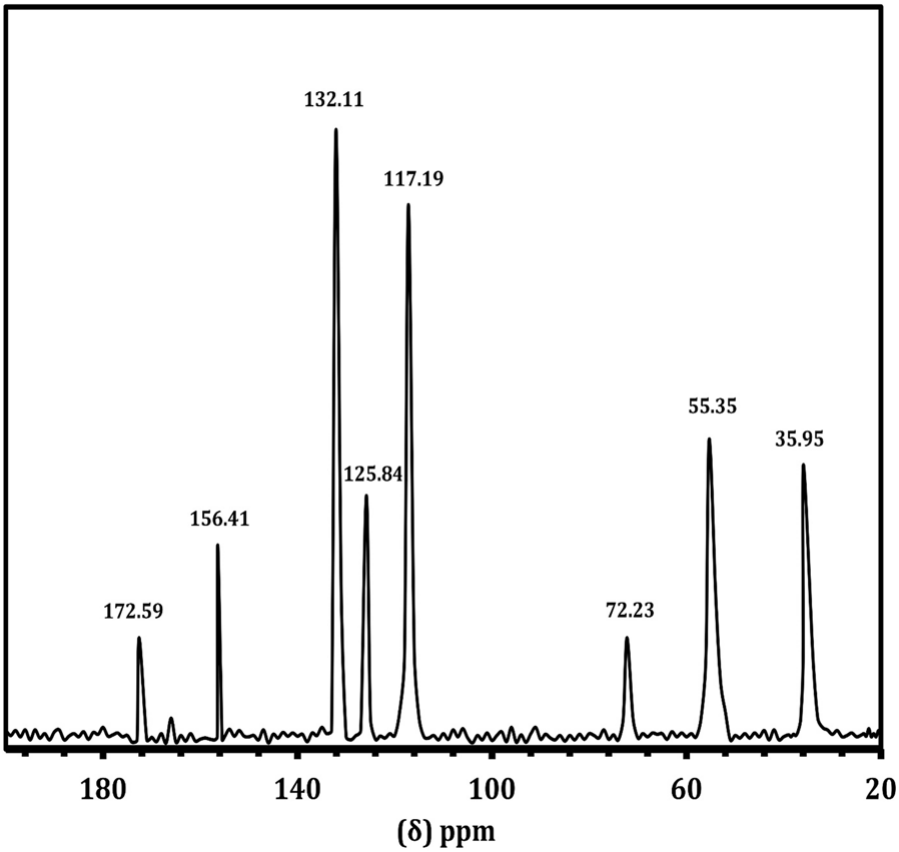
^13^C NMR spectrum of l-tyrosine extracted from *Rhizopus oryzae* strain AUMC14899

**Fig. 7.**
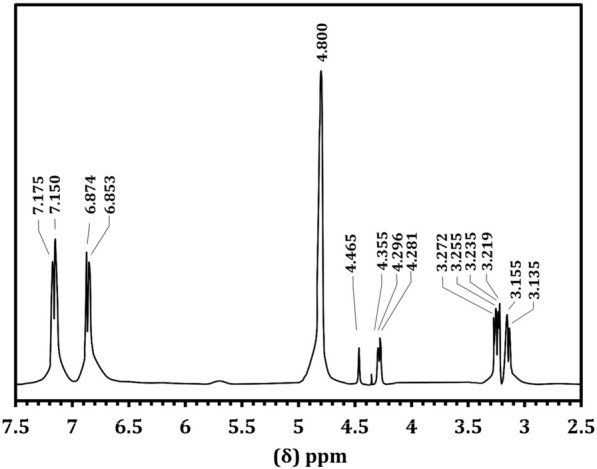
^1^H NMR spectrum of l-tyrosine extracted from *Rhizopus oryzae* strain AUMC14899

The NMR spectral analysis of LT is shown in Fig. 6. The carboxyl group gives its signal at 172.7 ppm. The aliphatic carbon carrying NH_3_^+^ and aromatic ring give their signals at 55.5 ppm and 35.9 ppm, respectively. The aromatic ring carbon produces its characteristic signal between 110 and 140 ppm. The aromatic ring carbon carrying the alkyl position gives its signal at 126.8 ppm. The ortho and meta carbons of the ring with an alkyl substituent give their signals at 132 and 117.2 ppm, respectively. The para carbon carrying on substitute shows its signal at 156.4 ppm. The proton NMR spectrum (Fig. 7) of the purified LT sample was recorded. The ortho hydrogen (proton) atoms in the aromatic structure can see each other as aligned (parallel) or opposed (antiparallel) and come to resonance twice. Thus, the ortho protons appear as doublets. These doublets have been seen at δ = 6.86 and δ = 7.16 ppm with the splitting factor of J = 8.4. The aliphatic CH proton has given its signal as triplet at δ = 4.27 ppm with the splitting factors of J = 5.6 and 7.2. The doublet signal at δ = 3.2 ppm is due to CH_2_. The multiple splitting is due to the presence of NH_3_ in the molecular structure.

**Fig. 8:**
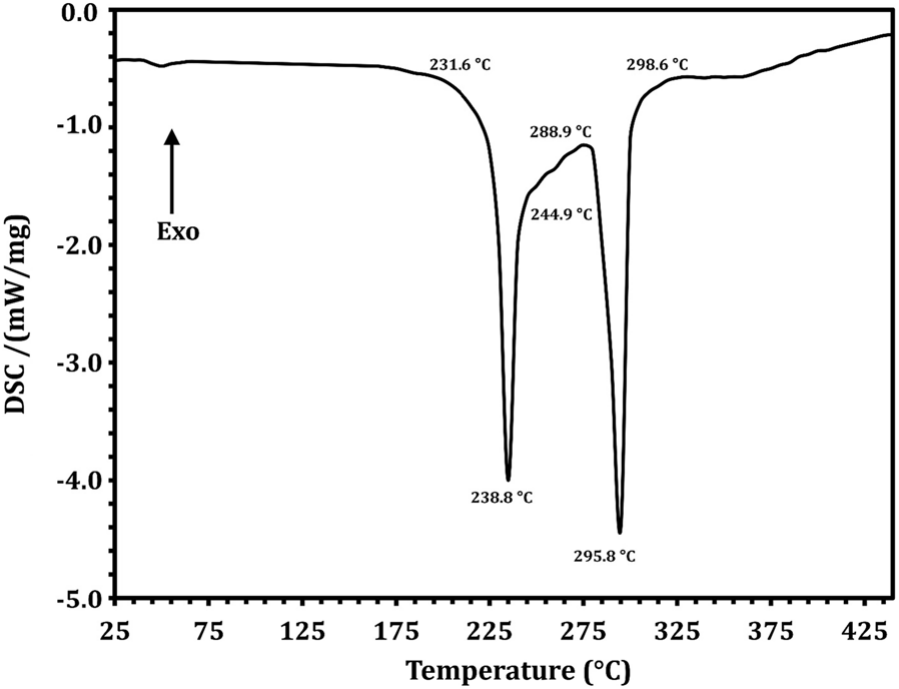
DSC spectrum of l-tyrosine extracted from *Rhizopus oryzae* strain AUMC14899

**Fig. 9:**
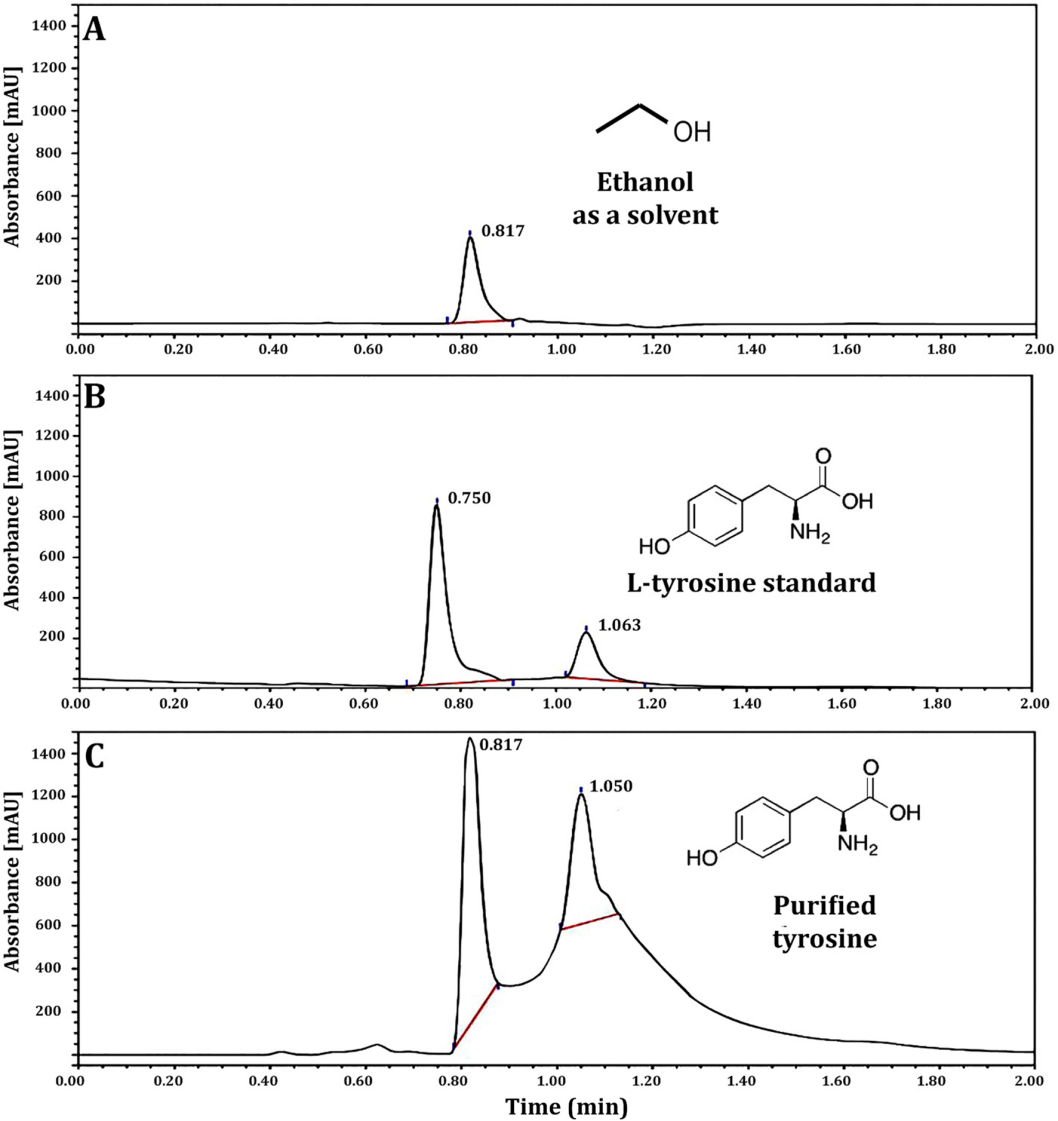
HPLC analysis of l-tyrosine extracted from *Rhizopus oryzae* strain AUMC14899. Ethanol (**A**). l-tyrosine standard (100 µg/ml) (**B**). Extracted l-tyrosine from *Rhizopus oryzae* strain AUMC14899 (275 µg/ml) (**C**)

The DSC thermogram of LT showed two endothermic peaks at 238.8 and 295.8 °C, and the melting started at 231.6 °C and the process completed at 244.9 °C (Fig. 8). A HPLC chromatogram confirmed the isolation and purification of LT by a peak at a retention time of 1.050 min compared to standard (Fig. 9). Figure 9A represents the blank (ethanol as solvent) to ensure selectivity and specificity of the method, while, Fig. 9B and C represented LT standard with a concentration of 100 µg/ml and purified LT with a concentration of 275 µg/ml, respectively. To the best of our knowledge, this is the first report to isolate and purify LT from endophytic *R. oryzae* from a cladode of *O. ficus-indica*.

### Antibacterial activity of LT

**Fig. 10:**
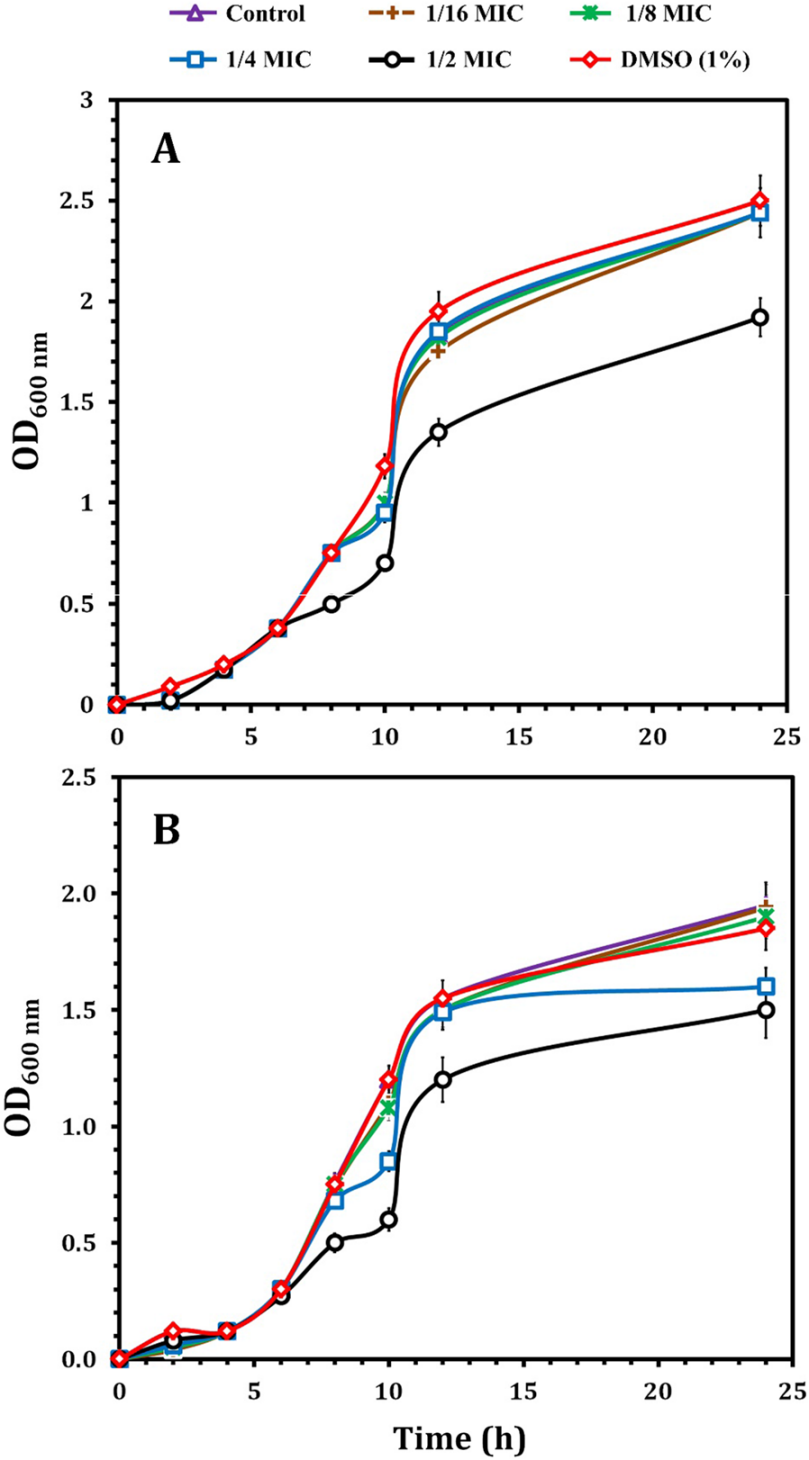
Growth curves for PA-02 (**A**) and SA-04 (**B**) strains in the presence of different concentrations of LT (1/2, 1/4, 1/8 and 1/16 MIC); DMSO (1%) served as negative control. These curves represent the average values of three reproducible experiments; OD: optical density; MIC: minimum inhibitory concentration

As depicted in Table 3, LT showed antibacterial activity against all the tested MDR bacterial strains with variable diameters of inhibition growth zones depending on bacterial species. MIC values of LT gave the lowest values of 6 µg/ml against PA-02 among gram-negative bacteria and 15 µg/ml against SA-04 among gram-positive bacteria. The growth curves of PA-02 and SA-04 at sub-MICs of LT over 24 h (Fig. 10). At 1/4, 1/8, and 1/16 × MIC, PA-02 exhibited the same development as the sample without LT. For SA-04, 1/8 × MIC and 1/16 MIC had no influence on bacterial growth. Nevertheless, LT demonstrated marginally slower growth of both strains during 24 h at 1/2 × MIC in contrast to DMSO, which had no impact on the bacterial growth of the tested strains.

**Table 3:** Antibacterial activity of l-tyrosine against six MDR bacterial strains

Strains	Zone of inhibition (mm) ± standard deviation					MIC (µg/ml)
	Different concentrations of l-tyrosine (µg/ml)					
	10	20	30	40	50	
Gram-negative bacteria						
PA-02	9.0 ± 0.5	11 ± 0.7	19 ± 0.3	28 ± 1.1	31 ± 1.0	6
PA-09	8.0 ± 0.7	10 ± 1.0	17 ± 0.9	25 ± 0.9	28 ± 1.5	8
KP-01	0.0 ± 0.0	8 ± 0.9	10 ± 0.4	16 ± 0.8	19 ± 0.5	15
EC-03	8.0 ± 1.0	11 ± 0.9	15 ± 1.0	20 ± 0.8	24 ± 1.1	10
Gram-positive bacteria						
SA-03	0.0 ± 0.0	9.0 ± 1.0	10 ± 0.5	13 ± 1.0	18 ± 1.0	20
SA-04	0.0 ± 0.0	8.0 ± 0.8	11 ± 0.6	19 ± 1.1	21 ± 0.9	15

MIC: Minimum inhibitory concentration. DMSO (1%) was used as a negative control and had no effect against the tested bacteria

### Effect of LT on biofilm production and bacterial viability

**Fig. 11:**
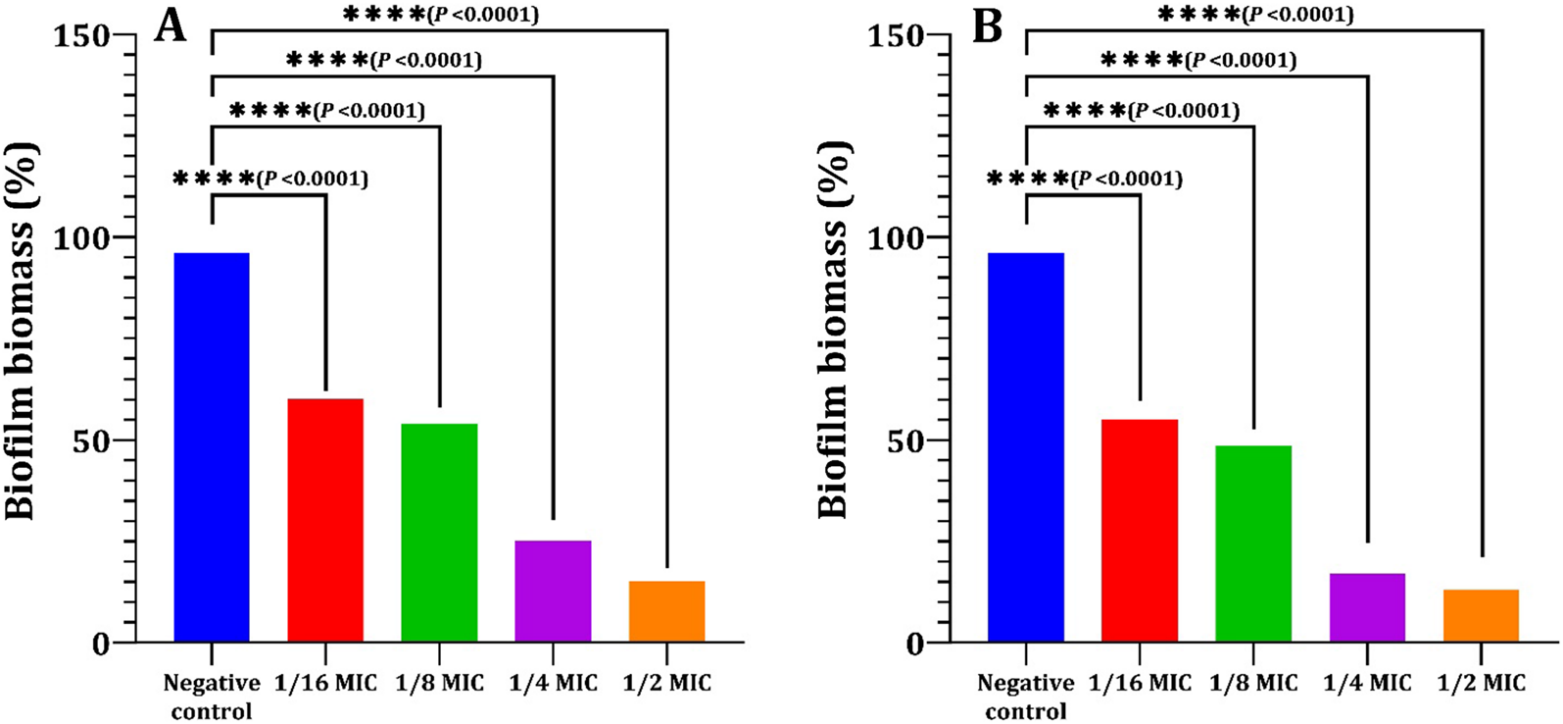
Inhibition of biofilm formation of PA-02 (**A**) and SA-04 (**B**) strains by l-tyrosine extracted from *Rhizopus oryzae* strain AUMC14899. Biofilm formation of both strains was significantly inhibited by sub-MICs concentrations (0.18–3 µg/ml and 0.93–7.5 µg/ml for PA-02 and SA-04, respectively) of l-tyrosine compared to negative control (bacteria grown without l-tyrosine). The values represent the mean of three independent replicates, with error bars representing the standard deviation of differences that are statistically significant at ^*^P ≤ 0.05, ^**^P ≤ 0.01, ^***^P ≤ 0.001, and ^****^P ≤ 0.0001, while *P* > 0.05 is non-significant (ns)

Results revealed that all tested strains were strong biofilm producers, except KP-01 and SA-03, which were moderate biofilm producers. Also, PA-02 and SA-04 were the most biofilm producers compared to others as in Table 4. The strains most capable of forming biofilms were discovered to be PA-02 and SA-04. As a result, we focused on the inhibitory effect of LT on these isolates' ability to form biofilms. The biofilm biomass was measured by the CV-staining assay as shown in Fig. 11 after treatment of PA–02 and SA–04 cells with LT at sub-MIC concentrations of 0.18–3 µg/ml and 0.93–7.5 µg/ml, respectively. All tested sub-MIC concentrations of LT significantly inhibited the biofilm formation of each strain at 24 h in a concentration dependent manner, as 1/2 × MIC of LT (3 µg/ml and 7.5 µg/ml for PA-02 and SA-04, respectively) significantly inhibited biofilm formation by 83% and 87%, respectively, compared to the negative control (*P* > 0.001).

**Table 4:** Biofilm production by the six MDR bacterial strains

Isolate code	(O.D)_570_ ± standard deviation	Biofilm production category
PA-02	0.81 ± 0.03	SP
PA-09	0.61 ± 0.01	SP
KP-01	0.41 ± 0.05	MP
EC-03	0. 60 ± 0.02	SP
SA-03	0.31 ± 0.08	MP
SA-04	0.75 ± 0.04	SP

SP: Strong biofilm producer (O. D > 4 × O.Dc); MP: Moderate biofilm producer (2 × O.Dc < O. D > 4 × O.Dc); PA: *P. aeruginosa*; KP: *K. pneumonia*; EC: *E. coli*; SA: *S. aureus*

**Fig. 12:**
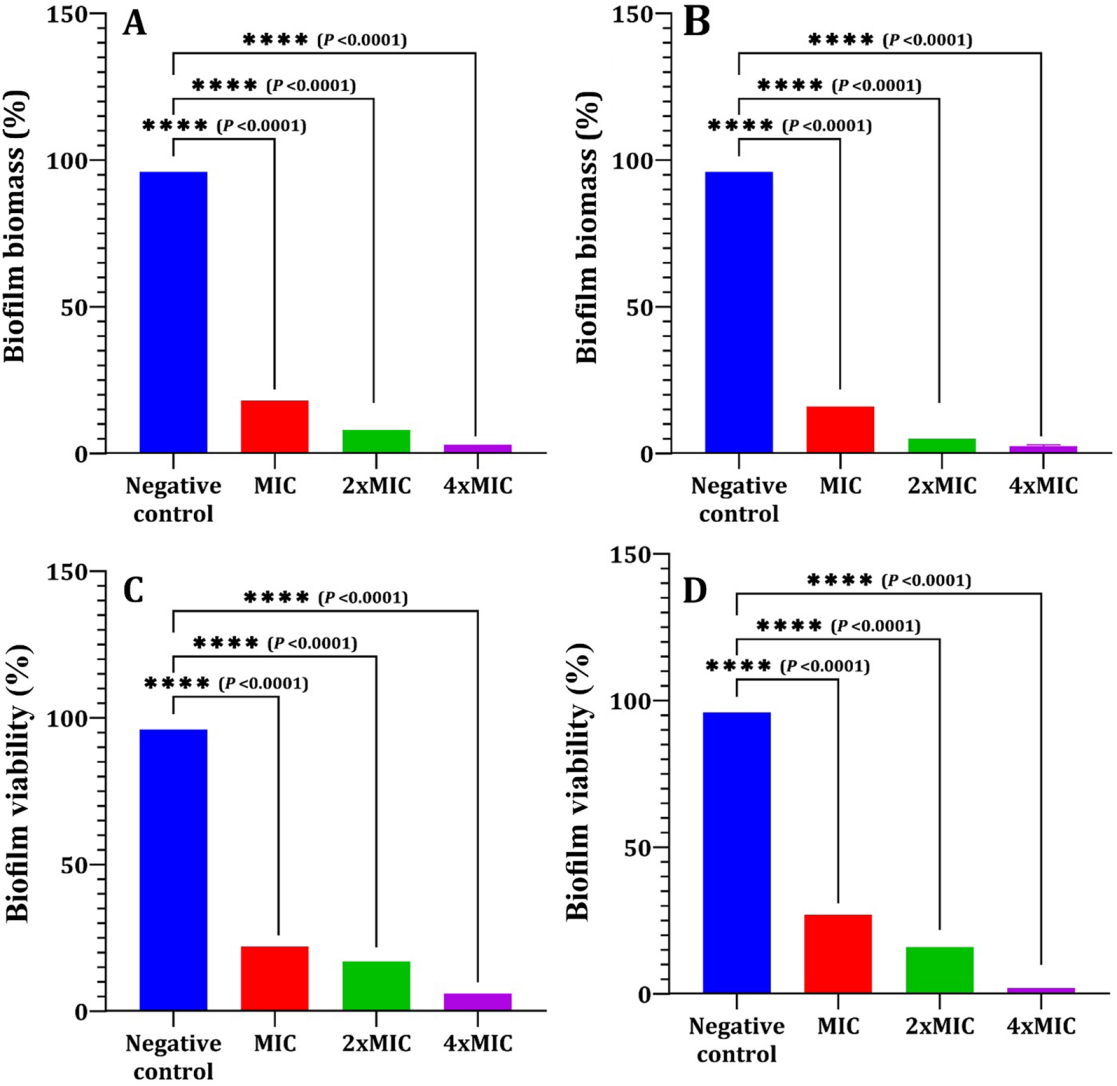
Eradicating effect of l-tyrosine on preformed biofilms of PA-02 and SA-04 strains. Biofilm biomass measured by crystal violet staining (**A**, **B**). Biofilm viability measured by XTT assay (**C&D**). The values represent the mean of three independent replicates, with error bars representing the standard deviation of differences that are statistically significant at ^*^P ≤ 0.05, ^**^P ≤ 0.01, ^***^P ≤ 0.001, and ^****^P ≤ 0.0001, while *P* > 0.05 is non-significant (ns)

Preformed biofilm and biofilm viability of LT against PA-02 and SA-04 strains were measured by CV staining and XTT assay, respectively. LT significantly reduced the amount of preformed biofilm and biofilm viability of both tested strains in a concentration dependent manner compared to the negative control (Fig. 12). Respectively, LT treatment at MIC, 2 × MIC and 4 × MIC values decreased biofilm biomasses by 80%, 88% and 93% for PA-02, and 85%, 92% and 97% for SA-04 (Fig. 12A and B). Moreover, LT reduced the viability of PA-02 and SA-04 biofilm cells by 66%, 80%, and 91%; and 72%, 84%, and 95%, respectively at MIC, 2 × MIC and 4 × MIC values for both strains (Fig. 12C and D).

**Fig. 13:**
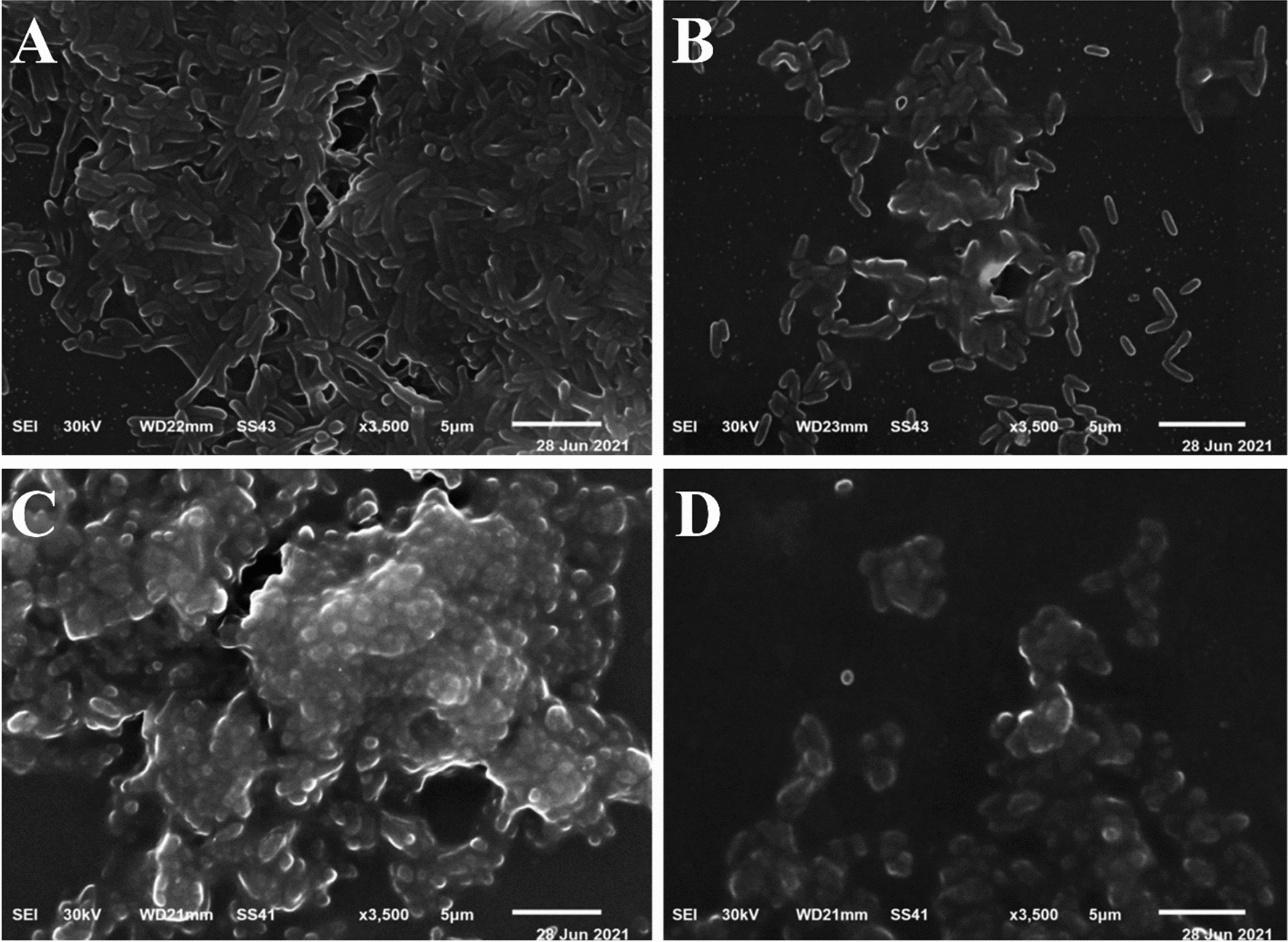
Representative SEM images for the effect of l-tyrosine (1/2 × MIC) on biofilm formation of PA-02 and SA-04 strains at ×3500 magnification, respectively. Control (**A**, **C**). Treated with l-tyrosine (**B**, **D**). Scale bar represents 1 μm

SEM was used to evaluate the effectiveness of LT against the biofilm development of selected strains (Fig. 13). PA-02 and SA-04 cells were incubated with or without 3 µg/ml and 7.5 µg/ml (1/2 × MIC) of LT, respectively. SEM images confirmed the inhibition of biofilm formation in both tested strains. Figure 13A, C revealed the typical multilayer growth of both control bacterial biofilms, while the biofilm was visibly reduced in LT-treated samples as shown in Fig. 13B and D. Moreover, fewer bacterial cells of both strains can be seen compared to control ones.

### Cytotoxicity and hemocompatibility of LT

Table 5 depicts the cytotoxic effect of purified LT on tumor and non-tumor cell lines. The findings showed that LT was relatively biocompatible because its IC_50_ values were greater than 500 µg/ml and it did not produce toxicity in non-tumor cell (HSF) cultures. The safety of LT was demonstrated by permitting normal cell development in healthy cultures and the absence of inhibition of the growth of non-tumor cell lines (IC_50_ > 500 µg/ml). On the contrary, LT had a greater toxic impact on the carcinoma cell line (A-431) with an inhibition percentage of 95.2% at a dosage of 500 µg/ml and an IC_50_ of 230 µg/ml.

**Table 5:** Cytotoxic potential of l-tyrosine

Compound	Conc (µg/ml)	Inhibition percentage (%)		IC_50_ (µg/ml)	
		HSF	A-431	HSF	A-431
l-tyrosine	0.5	1.4 ± 0.2	4.5 ± 0.4	> 500	230
	5	9.2 ± 0.2	11.4 ± 0.2		
	50	19.6 ± 0.3	20.1 ± 0.5		
	500	35.3 ± 0.2	90.2 ± 0.2		

HSF: human skin fibroblast; A-431: human epidermoid skin carcinoma; IC_50_: sample concentration at which cell growth was 50% inhibited

The hemolytic ratio of Triton X-100 was found to be 100% (Additional file 1: Fig. S3), while the hemolytic ratio of the LT was found to be equal to or < 2%, indicating that this newly isolated LT did not impact the integrity of the RBCs and included less than 5% of the biomaterial that was required by the international criteria.

## Discussion

Nowadays, endophytic fungi have great interest as they represent several bioactive compounds with promising biological activities [70]. The endophytic colonization of *O. ficus-indica* in our study in Egypt differed from the results found in other studies in Brazil, as most of the fungal genera obtained as endophytes of *O. ficus-indica* in Brazil were described as *Cladosporium cladosporioides* (20.43%) and *C. sphaerospermum* (15.99%) [71]. *Acremonium terricola*, *Monodictys castaneae*, *Penicillium glandicola*, *Phoma tropica* and *Tetraploa aristata*. However, among the fungi identified as endophytes of Egyptian *O. ficus-indica*, *R. oryzae* AUMC14899 was reported for the first time in our study. This variation in endophytes may be due to different habitats where organisms must adapt to various extreme environmental conditions; developing different physiological adaptation methods leads to a chemical variation, giving a chance to find novel compounds [72]. However, endophytic fungi include a variety of bioactive components, so it is necessary to purify and isolate these components using HPLC before testing them for antimicrobial activity [73]. The separation and HPLC fractionation of active components from endophytic *R. oryzae* isolated from *O. ficus-indica* are not documented, as far as is known. Therefore, this is the first study to isolate and purify LT from a new endophytic fungal isolate, *R. oryzae* AUMC14899 from *O. ficus-indica* as a new natural source for this amino acid. The isolation of LT detected in our endophytic isolate could be attributed to its high concentration in fungal extract. LT was separated and identified using TLC and HPLC, which were consistent with those of Li et al. [40] who used HPLC to isolate and identify amino acids from medicinal herbs. FTIR data showed that there were no hazardous cyano groups (C-N) or acetylenic groups (C–C), which supported the safety of LT [9]. NMR analysis showed only the characteristic peaks of LT that indicate the purity of the LT extracted from the tested fungus. Purified LT can retain texture up to 230 °C, according to DSC thermal analysis.

Amino acids can also be important drug candidates in the fight against antimicrobial resistance as well as antibiofilm agents [74]. As reported in several studies, amino acids that have various antibacterial activities also change the structure of the peptidoglycan layer interfere with protein synthesis or inhibit cell growth [75, 76]. Moreover, l-amino acids were found to inhibit biofilms of both gram-positive and gram-negative bacteria [77]. These are similar to our investigations, which demonstrated the strong antibacterial activity of LT against MDR-selected pathogens. Our data showed that LT showed MIC ranged from 6 to 20 µl against selected strains. These results were similar to the findings of Eugene-Osoikhia et al. [78], who demonstrated the same range of MIC against bacterial strains. The lower the MIC, the better in terms of activity [79]. Antimicrobial activity of natural compounds has been classified as good (MIC < 0.1 mg/ml), moderate (0.1 ≤ MIC ≤ 0.625 mg/ml) and weak (MIC > 0.625 mg/ml) [80]. Numerous antibiotics target the bacterial cell wall's peptididoglycan (PG) [81]. Additionally, antibacterial amino acids can target it [82]. Adding exogenous d-amino acids to bacteria during their production of PG throws off the amino acids' normal order and has a bactericidal impact [83]. Additionally, the exogenous amino acid glycine substitutes l-alanine at position 1 and d-alanine at positions 4 and 5 in PG, upsetting its normal sequence and inhibiting bacterial growth [84]. As a result, the antibacterial activity of LT may be caused by its integration into bacterial PG, which causes the growth of bacteria to be inhibited. Moreover, it has been proposed that the antibacterial action of LT may be caused by the presence of a phenolic group, which has a high inhibitory potential [85].

Free-living microorganisms attach to the surface of the host, and then create a biofilm that offers resistance to antimicrobial medications by protecting the associated cells residing inside of it [86]. To solve this issue, it is crucial to develop novel bioactive substances. In this study, LT acts as a prominent antibiofilm agent, inhibiting the growth of new biofilms and eliminating existing ones. At concentrations below the MIC, it was discovered that LT significantly prevented the development of biofilms in both the PA-02 and SA-04 bacteria (*P* < 0.001), however, it did not affect bacterial growth. Moreover, SEM images demonstrated a significant reduction in biofilm formation in treated strains with PLT compared with untreated ones. This suggests that the reduction in biofilm formation caused by LT was caused by its antibiofilm activity, not its antimicrobial activity. These results agree with other reports that LT derivatives have antibacterial and antibiofilm activities [78, 87].

Due to biofilm physical resistance to drug crossing and increased drug resistance, mature biofilms are more challenging to treat than those in the early stages [88]. As a consequence, LT at sub-MIC concentrations inhibited the growth of biofilms, while concentrations above those limiting biofilm formation on preformed biofilms were investigated, and it was found that LT significantly reduced the viability of both PA-02 and SA-04 biofilm cells at concentrations of MIC, 2 × MIC and 4 × MIC. These results were in agreement with Warraich et al. [89] who reported that both D and L aspartic and glutamic acids are able to inhibit and disperse *S. aureus* NCTC 8325 biofilms.

Numerous investigations examined the d-amino acids' antibiofilm mechanism. It has been observed that the inclusion of d-amino acids into peptidoglycan during its production alters the sequence of existing amino acids and the microfiber-microbial cell coupling breaks up, scattering the biofilm and liberating the sessile microbial cells [90]. However, the use of l-amino acids in preventing and dispersing microbial biofilms is not well understood. Therefore, further studies are needed to investigate the mechanism of the antibiofilm activity of LT.

One of the key characteristics of biomaterials utilized in wound healing applications is cell viability [91]. Regarding cytotoxicity, LT exhibited significant cytotoxicity against skin carcinoma A-431 cells, while LT appeared non-toxic against normal skin HSF cells. Numerous pathways may be used by this compound to cause cytotoxicity. The delocalization of the positive charge in the aromatic group is one potential way by which LT's cytotoxicity could be reduced [92]. Another mechanism that can reduce LT cytotoxicity is the presence of an amine group [85]. Furthermore, the type of cell is another way that could affect cytotoxicity [87].

The most frequently used initial toxicity assessment is hemolysis. For instance, a hydrogel wound dressing used in biomedical applications frequently comes into contact with blood. Therefore, any biomaterial that can speed up the healing of skin wounds must have appropriate levels of hemolytic activity [93]. The hemocompatibility of LT, as a novel candidate, must be taken into consideration for its biological safety in pharmaceutical and biomedical formulations. The findings of hemolytic activity, in this context, are consistent with those of other research showing that amino acids do not result in hemolysis, do not compromise the integrity of RBCs, and have great blood compatibility [69]. Another factor that might reduce the cytotoxicity is the great blood compatibility and lack of possible immunogenicity [94]. Therefore, in order to treat skin injuries, the hemocompatibility performance of LT may be promising. These results are the first published data suggesting LT as a promising safe anti-tumor and hemocompatiable agent that may be used in the biomedical field. Overall findings suggest that LT may have promising biomedical activities in recent years; pharmaceutical formulations have undergone substantial research in an effort to develop novel treatments for a variety of microbial diseases, including skin burn wounds.

## Conclusions

Endophytes are regarded as a great source of naturally occurring bioactive chemicals. Indeed, LT was produced by a new endophytic fungal isolate, *R. oryzae,* colonizing *O. ficus-indica* which grows in Egypt. To the best of our knowledge, this study may be the first to look into the biological performance of the newly isolated LT. The hemocompatibility and cytocompatibility results of LT showed promise for the pharmaceutical industry since they gave good and noticeable antibacterial activity against MDR bacterial pathogens with anti-biofilm activity. With a focus on battling MDR bacteria that cause skin burn infections, this study may therefore be a useful foundation for exploring LT as a new leading structure desired for pharmaceutical and biomedical therapies.

## Supplementary Information

**Additional file 1: Figure S1.** Plant material and collection. Collected stems of *Opuntia ficus-indica* (A). Area collection of Berket Al Sabaa along the train Road of Cairo‐Alexandria, Monufiya Governorate, Egypt (B). **Figure S2.** Thin layer chromatography for separation of l-tyrosine isolated from endophytic* R. oryzae. *Lanes 1-4: Isolated l-tyrosine from endophytic* R. oryzae*, Lane 5: Standard l-tyrosine. **Figure S3.** Hemolysis of LT.

## Data Availability

The datasets generated during and/or analysed during the current study are available from the corresponding author on reasonable request. All data generated or analysed during this study are included in this published article (and its additional information files).
