# Supplementary material for: Exploring the potential of Rhizopus oryzae AUMC14899 as a novel endophytic fungus for the production of l-tyrosine and its biomedical applications
Source: Microb Cell Fact. 2023 Feb 20;22:31. doi: 10.1186/s12934-023-02041-1 (PMC9942418; doi:10.1186/s12934-023-02041-1)
Supplement: Supplementary file 1 — Additional file 1: Figure S1. Plant material and collection. Collected stems of Opuntia ficus-indica (A). Area collection of Berket Al Sabaa along the train Road of Cairo‐Alexandria, Monufiya Governorate, Egypt (B). Figure S2. Thin layer chromatography for separation of l-tyrosine isolated from endophytic R. oryzae. Lanes 1-4: Isolated l-tyrosine from endophytic R. oryzae, Lane 5: Standard l-tyrosine. Figure S3. Hemolysis of LT. [file 12934_2023_2041_MOESM1_ESM.docx]

**Additional file**

**
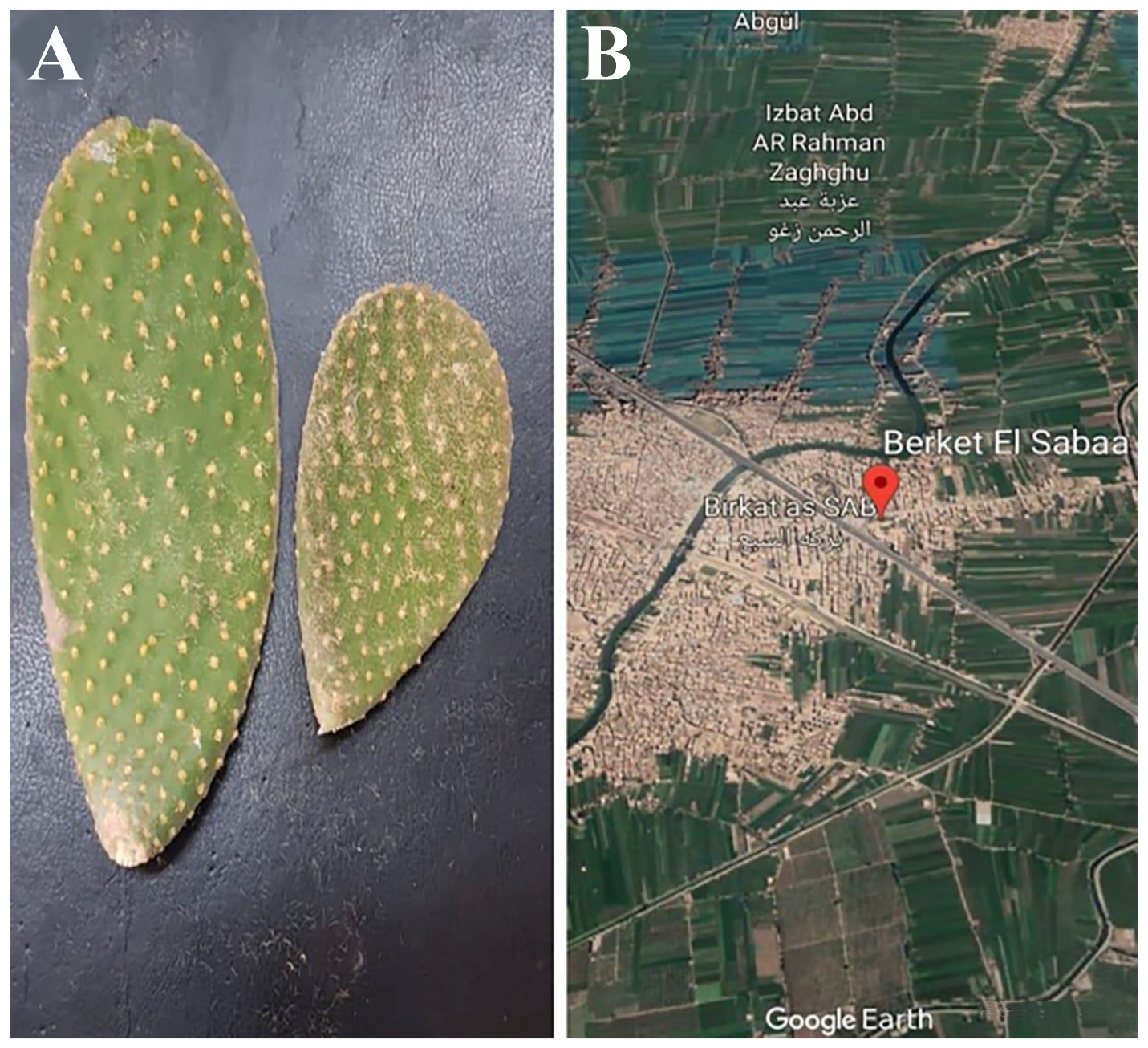
**

**Figure S1.** Plant material and collection. Collected stems of *Opuntia ficus-indica* (**A**). Area collection of Berket Al Sabaa along the train Road of Cairo‐Alexandria, Monufiya Governorate, Egypt (**B**).

**
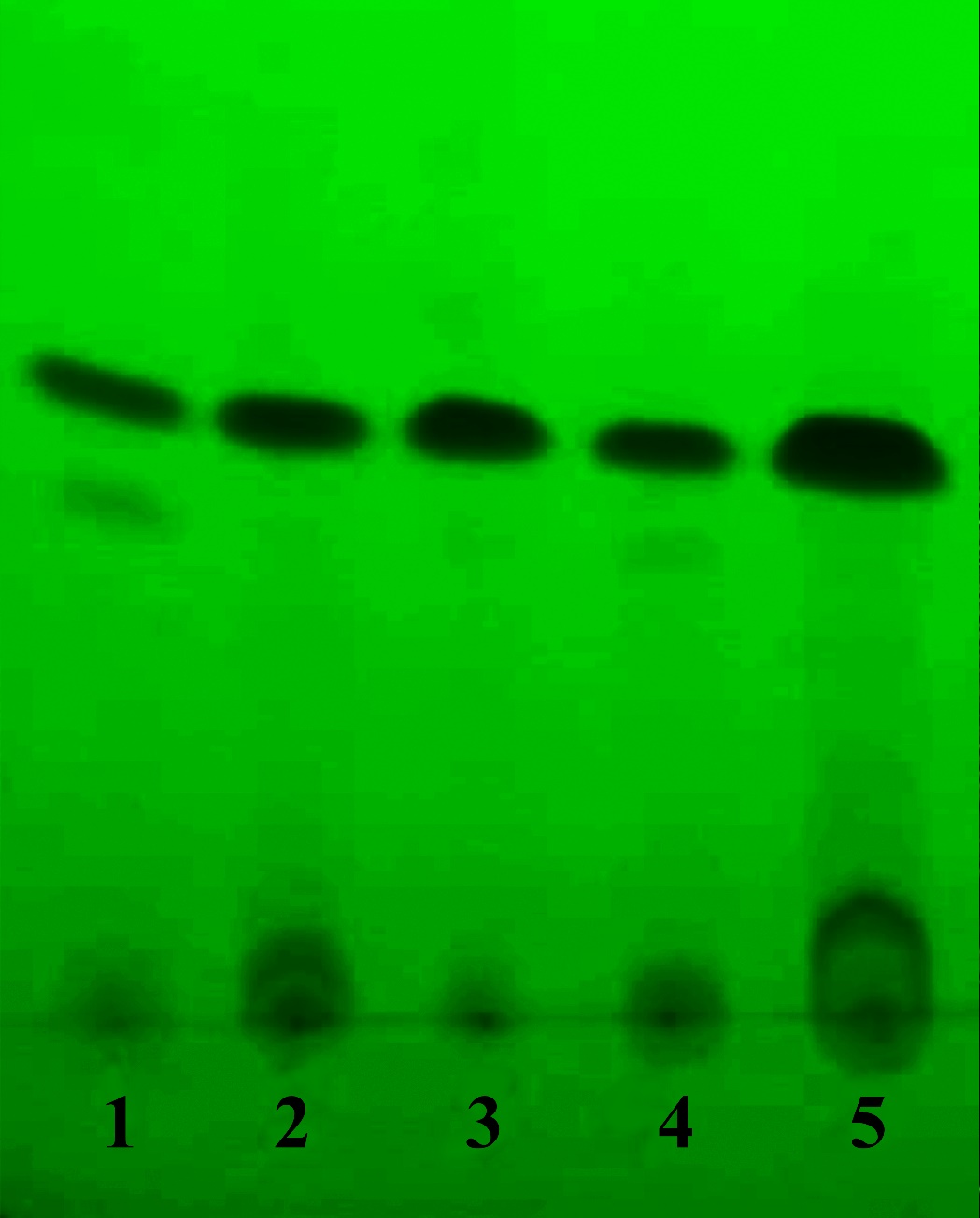
**

**Figure S2.** Thin layer chromatography for separation of L-tyrosine isolated from endophytic *R. oryzae.* Lanes 1-4: Isolated L-tyrosine from endophytic *R. oryzae*, Lane 5: Standard L- tyrosine.

**
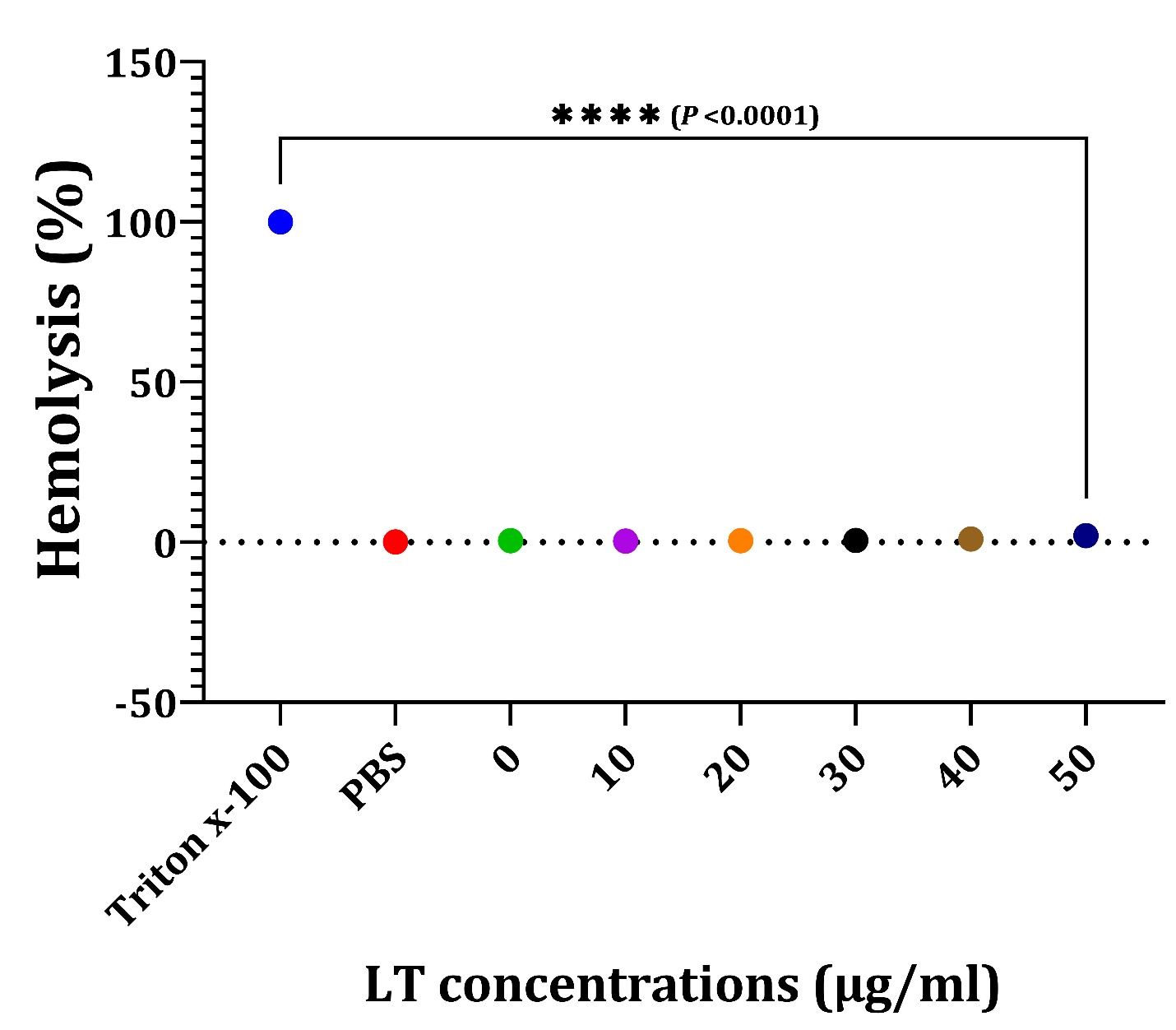
**

**Figure S3.** Hemolysis of LT.
